# Frequent internet use is associated with better episodic memory performance

**DOI:** 10.1038/s41598-024-75788-1

**Published:** 2024-10-22

**Authors:** Weixi Kang, Antonio Malvaso

**Affiliations:** 1 School of Arts and Humanities, Tung Wah College, Cheung Kung Hai Memorial Building, 90A Shantung Street, Mong Kok, Kowloon, Hong Kong, China; 2 Department of Brain Sciencies, Imperial College, London, United Kingdom; 3 Department of Brain and Behavioral Sciences, University of Pavia, Pavia, Italy

**Keywords:** Transactive memory, Internet use, Episodic memory, Immediate word recall, Delayed word recall, Psychology, Human behaviour

## Abstract

As the internet is becoming more and more accessible and prevalent, there has been growing interest in determining the effect of internet use on human cognition, particularly memory. However, much less is known about how internet use frequency is related to episodic memory, which refers to the memory of past events as opposed to facts (i.e., semantic memory). Episodic memory is very relevant to the internet because of the notion that the internet is a form of transactive memory, which requires people to retrieve how information was accessed (i.e., episodic memory) rather than the information itself (i.e., semantic memory). By analyzing data from 36, 542 participants with 44.06% females and a mean age of 46.63 years old from the Understanding Society using multivariate and univariate analysis of variances (ANOVA), the current study found that the frequency of internet use is positively related to episodic memory (assessed using the immediate recall and delayed word recall tasks). These results provide support to the notion that the internet is a form of transactive memory and the “use it, or lose it” theory.

## Introduction

To respond to the question “How has the internet changed your life?”, people normally responded that the internet can help find new friends, renew old friendships, build romantic relationships, seek career furthering career opportunities, travel, and shopping^1^. However, most people respond that the internet has changed the information that can be accessed^1^. Indeed, most people from the developed world have access to all kinds of existing information with their fingers^2^. However, although important, few studies have investigated how internet use might relate to human cognition such as episodic memory^2^.

### Semantic vs. episodic memory

Tulving^3^ proposed that memory research can be benefited from distinguishing semantic and episodic memory. Tulving^3^ defined episodic memory as knowledge “*about temporally dated episodes or events*,* and temporal-spatial relations among these events*” and pointed out that episodic memory is kept “*in terms of its autobiographical reference to the already existing contents of the episodic memory store.*” On the other hand, semantic memory is defined as the “*memory necessary for the use of language. It is a mental thesaurus*,* organized knowledge a person possesses about words and other verbal symbols*,* their meaning*,* and referents*,* about relations among them*,* and about the rules*,* formulas*,* and algorithms for the manipulation of these symbols*,* concepts*,* and relations*”^3^.

### Internet as transactive memory

Along with the obvious advantages brought by the internet, the internet age has also introduced the possibility of replacing the human memory system with the internet, especially for semantic memory (i.e., the memory of facts), which is independent of other types of memory in the neural system^4^. For instance, Sparrow et al.^5^ provided evidence regarding how internet information affects typical memory processes by demonstrating that people who access the internet are more likely to remember the way the information can be accessed rather than the information itself. This suggests that people are increasingly reliant on the internet for retrieving information.

One might argue that this is not a unique case for the internet, but just an example of external memory or transactive memory^5,6^, which refers to the way people choose to source external information, such as other individuals, tools, and technologies, so that they can just remember the source of the information rather than the information itself^6^. Although transactive memory may be beneficial at a group level, it has been proposed that using transactive memory systems reduces one’s ability to recollect specifics of the externally stored information^7^. This can be explained by the fact that people typically use transactive memory for the purpose of cognitive offloading, which implicitly reduces the allocation of cognitive resources, as they know this information is stored externally for future use. This phenomenon has been shown in various contexts, including those of teamwork^8^ and other “non-internet” technologies (e.g., photos that help people to offload their memory of the objects being photographed;^9^).

However, the internet is something novel and different from other transactive memory systems^10,11^. Importantly, the internet goes beyond the transactional aspect and is different from other types of cognitive offloading in two ways. First, the internet does not place any responsibility on the user to maintain information for others to reference later on^11^. Second, the internet is a single entity that is in charge of storing and recalling all information virtually, which does not require its users to remember exactly where the information is located or what is stored. Thus, the internet is becoming a supernormal stimulus^10^ for transactive memory, making other forms of cognitive offloading (e.g., other individuals, tools, technologies) redundant, as they are outcompeted by the internet’s ability to store and retrieve external information.

### Internet use and episodic memory

Episodic memory is adaptive, so its function is not only about record-keeping of events that have happened but can offer present and future adaptation^12–15^. From an evolutionary point of view, making plans for one’s personal future based on memories of past events is an important advantage^12,14^. As surveyed above, the internet acts as a transactive memory system so that people remember better their way of retrieving formation rather than the information itself. Thus, episodic memory must be involved in such a process. Specifically, when one tries to retrieve the details of cooking a dish from a previously watched cooking tutorial online, one must retrieve the details in terms of how he or she found such a cooking tutorial.

### The current study

Thus, as reviewed, internet use is closely related to memory. However, previous studies only looked at the short-term effect of internet use and other types of memory rather than semantic memory in small sample-size experimental studies, much less is known about how internet use in daily life could affect episodic memory in a nationally representative sample from the UK. The current study hypothesizes that internet use frequency is positively related to episodic memory because frequent use of the internet may enhance the ability to remember the way information can be accessed (e.g., a more episodic form of memory). According to theories such as the “use it or lose it” theory^16,17^, which suggests that continuously using one ability may improve that ability, frequent use of episodic memory may then in turn relate to better episodic memory ability. This is because the internet users tend to remember the way of accessing the information (episodic memory) rather than the information itself (semantic memory). The “use it or lose it” theory supports this hypothesis by implying that regular engagement with tasks that exercise episodic memory (such as internet use) strengthens the neural pathways associated with episodic memory, leading to improved cognitive function in this area over time.

## Method

### Data

Data were used from Wave 3 Understanding Society: the UK Household Longitudinal Study (UKHLS), which was collected between 2011 and 2012^18^. Data collection was approved by the University of Essex Ethics Committee. The UKHLS was previously known as The British Household Panel Study (BHPS). There were 36, 542 participants with 44.06% females and a mean age of 46.63 (± 18.32) years old left after removing missing variables of interest (Table 1).

**Table 1:** Descriptive statistics for demographic variables, internet use frequency, and the immediate and delayed recall task performance.

Variable	Mean	S.D.
Age	46.63	18.32
Immediate word recall	9.24	1.73
Delayed word recall	5.21	2.08
	N	%
Sex		
Male	16,102	44.06
Female	20,440	55.94
Monthly income		
Less than £1000	15,667	42.87
More than £1000 but less than £2000	14,139	38.69
More than £2000	3736	18.43
Highest educational qualification		
Below college	25,906	70.89
College	10,636	29.11
Legal marital status		
Single	17,778	48.65
Married	18,764	51.35
Residence		
Urban	28,310	77.47
Rural	8232	22.53
Internet use frequency		
Everyday	20,432	55.91
Several times a week	6381	17.29
Several times a month	1842	5.04
Several times a year	1327	3.63
Never use	6623	18.12

### Measures

#### Internet use frequency (independent variable)

Participants indicated the frequency of internet use using options including “Everyday,” “Several times a week,” “Several times a month,” “Once a month,” “Less than once a month,” “Never use,” and “No access at home, at work or elsewhere.” “Once a month,” and “Less than once a month” were combined into a single category “Several times a year.” “No access at home, at work, or elsewhere” was combined with the “Never use” category.

#### The immediate and delayed recall task (dependent variables)

Based on a description from UKHLS, “For this task, the computer reads a list of 10 words to standardise the presentation and speed of the word list. The interviewer checks if the respondent can hear the computer playing a short test message. If the voice cannot be heard the interviewer checks again following adjustment of the volume. If the respondent still cannot hear the computer’s voice, the interviewer reads the words at a slow steady rate of about one word every two seconds. The list of words is not repeated. No aids are allowed for the test. Interviewer: The computer will now read a set of 10 words. I would like you to remember as many as you can. We have purposely made the list long so it will be difficult for anyone to remember all the words. Most people remember just a few. Please listen carefully to the set of words as they cannot be repeated. When it has finished, I will ask you to recall aloud as many of the words as you can, in any order. Is this clear? Now please tell me the words you can remember. Respondents give the words in any order. The interviewer codes each correct response. For the delayed word recall test, after the Number Series test (below), respondents were again asked to remember the words from the list. The interviewer codes each correct response”^19^. Both the immediate and delayed recall task scores were standardized before analysis (mean = 0, SD = 1) so results from the multiple comparison tests are in Cohen’s d units.

#### Demographics (control variables)

Control variables include age, sex, monthly income, highest educational qualification, marital status, and residence. Age was used as it is. Sex was coded as male vs. female. Monthly income was coded as monthly income < = 1000 vs. 1000 & monthly income < = 2000 vs. monthly income > 2000. The highest educational qualification was considered as below college vs. college. Marital status was regarded as single vs. married/civil partner vs. divorced/separated. Residence was coded as urban vs. rural.

### Analysis

A multivariate analysis of variance (MANOVA) was used to analyze the data respectively to determine if there was a main effect of internet use frequency on episodic memory despite the type of task while taking demographics into account. A MANOVA should be used prior to univariate ANOVA because it considers multiple dependent variables simultaneously, thereby accounting for the potential correlations between them. This multivariate approach helps to control for Type I errors that could arise from conducting multiple univariate ANOVAs independently, as each separate test increases the risk of falsely finding significant results due to chance. Additionally, MANOVA can reveal multivariate effects that univariate ANOVAs might miss, providing a more comprehensive understanding of the data. If MANOVA indicates significant differences, it justifies further exploration with univariate ANOVAs to identify which specific dependent variables contribute to the overall effect. A univariate ANOVA then was applied to determine the effect of the internet use frequency condition on each of the recall tests. A multiple comparison test following Turkey’s honestly significant difference procedure^20^ was to compare the differences between internet use frequency groups. All analyses were conducted using a customized script on MATLAB 2018a.

## Results

The current study found that internet use frequency had a significant multivariate effect on episodic memory (F(1,36445) = 378.84, *p* < 0.001) after taking into account demographics. Moreover, there were also significant univariate effects for the immediate recall task (F(4, 36568) = 341.73, *P* < 0.001), and the delayed recall task (F(4,36445) = 267.25, *P* < 0.001) after controlling for demographics (Table 2).

**Table 2:** The ANOVA results by taking internet use frequency and demographics as independent variables and the immediate recall task and delayed recall task as the dependent variable respectively.

Variable	Immediate word recall						Delayed word recall					
	Sum Sq.	d.f.	Mean Sq.	F	p	η2	Sum Sq.	d.f.	Mean Sq.	F	p	η2
Age	1373.5	86	15.97	21.78	< 0.001	0.04	1677.61	86	19.51	26.24	< 0.001	0.05
Sex	258.7	1	258.73	352.87	< 0.001	0.01	293.2	1	293.20	394.47	< 0.001	0.01
Monthly income	152.2	2	76.09	103.78	< 0.001	0.00	107.4	2	53.70	72.25	< 0.001	0.00
Education	483.4	1	483.43	659.34	< 0.001	0.01	388.55	1	388.55	522.75	< 0.001	0.01
Marital Status	2.3	1	2.25	3.07	0.08	0.00	2.92	1	2.92	3.93	< 0.05	0.00
Residence	68.9	1	68.95	94.04	< 0.001	0.00	59.26	1	59.26	79.73	< 0.001	0.00
Internet use frequency	1002.2	4	250.56	341.73	< 0.001	0.03	794.58	4	198.64	267.25	< 0.001	0.02
Error	26721.4	36,445	0.73				27088.95	36,445				
Total	34153.5	36,541					34381.96	36,541				

Multiple comparison tests revealed that everyday internet users have better immediate word recall performance than participants who are in all other groups. They had better immediate word recall performance than people who used the internet several times a week (standardized mean difference (i.e., Cohen’s d) = 0.07, [95% C.I.: 0.04, 0.10], *p* < 0.001), several times a month (standardized mean difference = 0.16, [95% C.I.: 0.10, 0.22], *p* < 0.001), several times a year (standardized mean difference = 0.29, [95% C.I.: 0.22, 0.36], *p* < 0.001), and those who never use the internet (standardized mean difference = 0.55, [95% C.I.: 0.51, 0.59], *p* < 0.001). Similarly, people who used the internet several times a week performed better in immediate word recall tests than people who used the internet several times a month (standardized mean difference = 0.16, [95% C.I.: 0.05, 0.27], *p* = 0.001), several times a year (standardized mean difference = 0.22, [95% C.I.: 0.28, 0.53], *p* < 0.001), and those who never used the internet (standardized mean difference = 0.48, [95% C.I.: 0.43, 0.52], *p* < 0.001). People who used the internet several times a month had better immediate word recall test scores than people who used the internet several times a year (standardized mean difference = 0.13, [95% C.I.: 0.05, 0.21], *p* < 0.001), and those who never used the internet (standardized mean difference = 0.39, [95% C.I.: 0.33, 0.45], *p* < 0.001). People who used the internet several times a year had better performance than people who never used the internet (standardized mean difference = 0.26, [95% C.I.: 0.19, 0.33], *p* < 0.001).

**Fig. 1:**
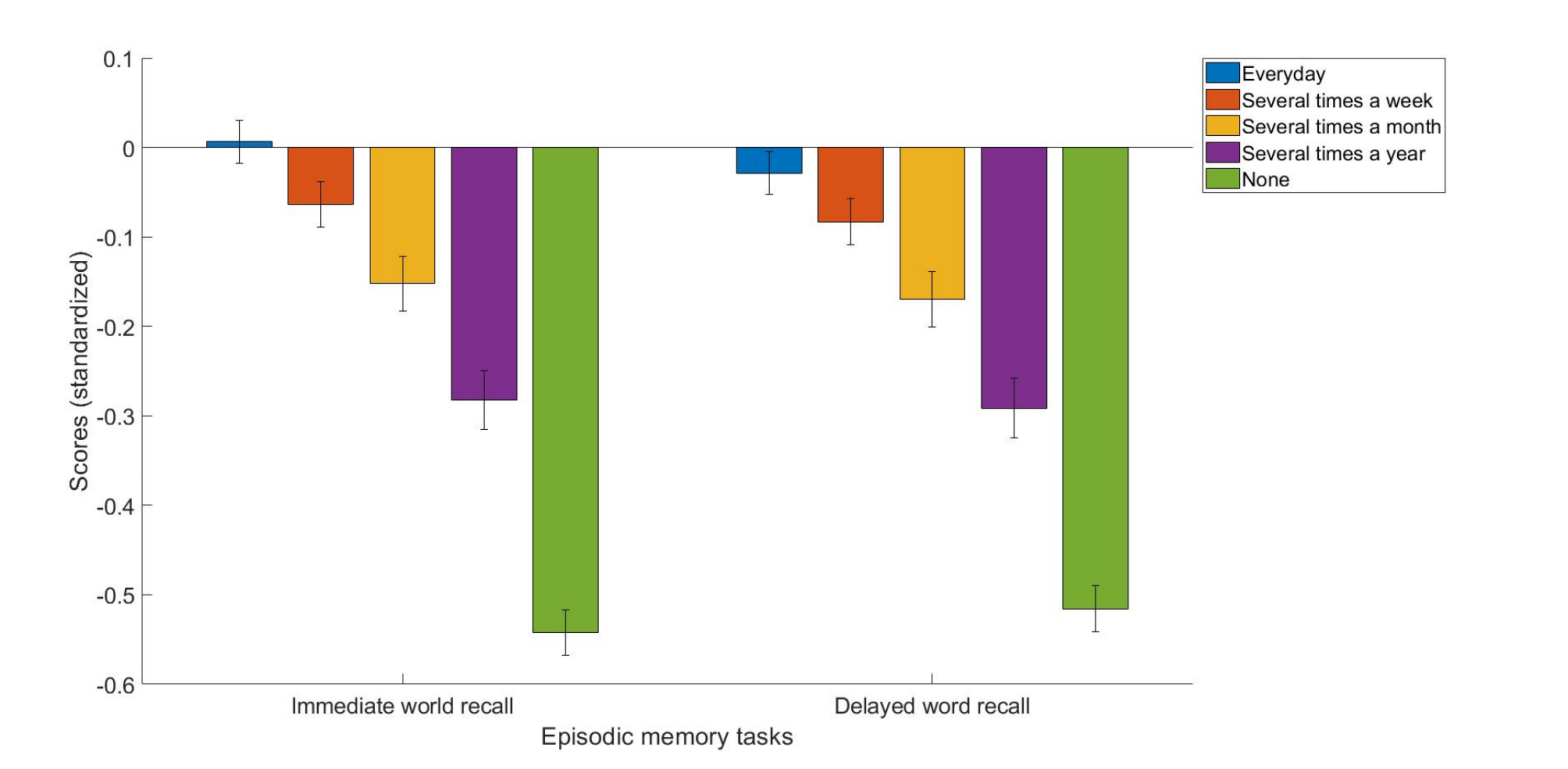
The standardized mean scores for the immediate and delayed word recall task across the internet use frequency groups with standard error.

As for the delayed word recall test, daily internet users had better performance than people who used the internet several times a week (standardized mean difference = 0.05, [95% C.I.: 0.02, 0.09], *p* < 0.001), several times a month (standardized mean difference = 0.14, [95% C.I.: 0.08, 0.20], *p* < 0.001), several times a year (standardized mean difference = 0.26, [95% C.I.: 0.19, 0.33], *p* < 0.001), and those who never used the internet (standardized mean difference = 0.49, [95% C.I.: 0.45, 0.53], *p* < 0.001). People who used the internet several times a week had better performance than people who used the internet several times a month (standardized mean difference = 0.09, [95% C.I.: 0.02, 0.15], *p* < 0.01), several times a year (standardized mean difference = 0.21, [95% C.I.: 0.14, 0.28], *p* < 0.001), and those who never used the internet (standardized mean difference = 0.43, [95% C.I.: 0.39, 0.48], *p* < 0.001). People who used the internet several times a month had better delayed word recall test scores than those who used the internet several times a year (standardized mean difference = 0.12, [95% C.I.: 0.04, 0.21], *p* < 0.001) and those who never used the internet (standardized mean difference = 0.34, [95% C.I.: 0.28, 0.41], *p* < 0.001). Finally, people who used the internet several times a year had better delayed word recall performance than those who never used the internet (standardized mean difference = 0.22, [95% C.I.: 0.15, 0.30], *p* < 0.001). Means and standard errors of the immediate and delayed word recall test scores for each internet use group were plotted in Fig. 1.

## Discussion

Episodic memory plays a critical role in the transactive memory associated with the internet. The objective of the current research is to investigate how internet frequency is associated with episodic memory using the immediate and delayed word recall tasks. The current study hypothesized that internet use frequency is positively associated with episodic memory. By analyzing data from the UKHLS, the current study found that the hypothesis of the current study is supported by the current results. Implications and limitations will be discussed.

The current results suggested that there was both multivariate and univariate effect of internet use frequency on the immediate and delayed memory recall tasks as indicated by results from both the multivariate and univariate ANOVA. MANOVA assesses multiple dependent variables simultaneously, revealing whether there are significant differences in a combination of these variables across groups, thereby considering their intercorrelations and providing a more holistic view than univariate ANOVAs. While univariate ANOVAs evaluate each dependent variable independently, potentially increasing the risk of Type I errors, MANOVA controls for this by testing the dependent variables together. In the context of current research, MANOVA showed significance, it suggests an overall effect of internet use on both the immediate recall and delayed recall tasks, offers more robust evidence regarding the associations between internet use and episodic memory in addition to univariate ANOVA. This approach offers a deeper understanding of the multivariate relationships and overall patterns, thus identifying subtle effects of internet use and interactions between the immediate and delayed word recall task that could be missed when analyzed individually.

Multiple comparison test results also indicated that people who used the internet more frequently had higher task performance than people who used the internet less frequently. Moreover, these effects were consistently found in different internet use frequency groups and across tasks. These pieces of evidence support the claim that internet use is related to episodic memory, the more frequently internet usage the better episodic memory one would expect.

These findings are generally consistent with the notion that the internet can serve as transactive memory^21^. Episodic memory comes into play when one wants to recall where the external information is stored. Thus, although semantic memory can be negatively affected by internet use (e.g^22–24^), internet use is positively associated with episodic memory given that more frequent use of the episodic form of memory promotes it (i.e., “use it or lose it” theory;^16,17^). The results of the current study agree with the adaptive function of episodic memory, which is not only in record-keeping of the past but can offer present and future fitness^12–15^. Episodic memory allows individuals to mentally time-travel, enabling them to reconstruct past experiences and simulate potential future scenarios. This capacity for mental simulation is crucial for planning, problem-solving, and decision-making. By leveraging past experiences, individuals can anticipate future needs and challenges, enhancing their ability to navigate complex environments and make informed choices. Therefore, the possible enhancement of episodic memory through internet use suggests that digital environments, by requiring users to remember and locate stored information, may foster cognitive processes that are beneficial for both present functioning and future planning.

More specifically, immediate and delayed memory scores being related to internet use frequency can be attributed to the varying cognitive demands and stimulation associated with internet activities. Immediate memory, which involves the ability to recall information shortly after it is presented, might be enhanced by frequent internet use due to the constant need to quickly process and respond to information, such as reading articles, browsing social media, or engaging in online conversations. These activities can stimulate short-term recall and information-processing skills. On the other hand, delayed memory, which involves recalling information after a longer period, may also be influenced by internet use, albeit differently. Frequent internet users often engage in activities that require them to remember information over time, such as returning to read saved articles, recalling instructions or strategies from online tutorials, or revisiting discussions from forums. The habitual practice of these cognitive tasks could enhance both immediate and delayed memory performance, reflecting a broader cognitive engagement facilitated by regular internet use.

The current research has several limitations. First, the cross-sectional design cannot establish a causal relationship between internet use and episodic memory. It remains unclear whether frequent internet use improves episodic memory or if individuals with better episodic memory use the internet more. Future studies should consider a longitudinal design to clarify this relationship.

Additionally, Firth et al.^21^ highlight that internet use serves two primary purposes: social communication and information consumption. Differentiating these purposes could enhance our understanding of how internet use impacts memory. Studies suggest that online social interactions affect cognition similarly to real-world socialization, and the size of online social networks correlates with associative memory^25^. Excessive social media use has been linked to decreased gray matter volume in brain regions related to social cognition, emotional regulation, and addiction^26,27^. As Firth^21^ noted, social and non-social internet contexts are interconnected. For example, information gained in non-social contexts often shapes social interactions. Future research should explore the interplay between these contexts and their effects on cognition. It is also important to examine how different platforms like Instagram, Facebook, and Snapchat affect episodic memory. While social interactions can be beneficial, extensive internet use might reduce real-world interactions and disrupt behaviors like sleep, potentially impacting memory^28^. Further research should investigate the real-time effects of internet use on brain regions supporting memory, and how different types of internet use—such as transactive memory—might influence episodic memory. Lastly, future studies should explore potential links between internet use and other types of memory, such as semantic memory, and its effects on broader cognitive domains.

There are several implications that can be drawn from the current research. Health professionals and organizations can come up with evidence-based guidelines on amounts and types of internet usage similar to public health guidelines for other health behaviors, such as sleep and physical activities^29,30^. These guidelines can then be spread across the population so that individuals can make decisions based on these guidelines regarding their internet use. Moreover, given internet use is associated with better episodic memory, future studies could also investigate how internet technologies can be utilized to prevent and maybe improve episodic memory in people with or in the progress of episodic dementia. Indeed, there have been examples of using internet technology to improve human health and cognition. For instance, the most long-lasting example is internet-delivered cognitive behavioral therapy (iCBT), which can deliver CHT through computerized technology to reduce people with various psychological symptoms. Moreover, it has been shown to be as effective as traditional face-to-face therapy^31^. Smartphone technologies also create novel platforms to deliver user-friendly psychological interventions based on the internet^32^. One meta-analysis showed the efficacy of using smartphone-delivered to reduce both depression and anxiety^33^. However, to what extent these effects are due to the therapy itself rather than using the smartphones and individuals’ connection with their smartphones has to be determined^34^.

In conclusion, the current study investigated the relationship between internet use frequency and episodic memory association in a large cohort of nationally representative samples from the UK. Although the current study provided some novel findings regarding how internet use frequency may relate to episodic memory, future studies are needed to explore further the other types of memory and how the association can be affected in different contexts (social vs. nonsocial) and platforms, and how they relate to brain structure and functions. Finally, the current research can be utilized as empirical evidence for developing public health guidelines and digital therapy for people with psychological and neurological conditions such as episodic dementia.

## Data Availability

Publicly available datasets were analyzed in this study. This data can be found here: https://www.understandingsociety.ac.uk.

## References

[1] Colley, A. & Maltby, J. Impact of the internet on our lives: Male and female personal perspectives. *Comput. Hum. Behav.* **24**(5), 2005–2013 (2008).

[2] Firth, J. et al. The online brain: How the internet may be changing our cognition. *World Psychiatry*. **18**(2), 119–129 (2019). 31059635 10.1002/wps.20617 PMC6502424

[3] Tulving, E. 12. Episodic and semantic memory. In *Organization of Memory* (eds Tulving, E. & Donaldson, W.) 381–403 (Academic Press, 1972).

[4] Vargha-Khadem, F. et al. Differential effects of early hippocampal pathology on episodic and semantic memory. *Science*. **277**(5324), 376–380 (1997). 9219696 10.1126/science.277.5324.376

[5] Sparrow, B., Liu, J. & Wegner, D. M. Google effects on memory: Cognitive consequences of having information at our fingertips. *Science*. **333**(6043), 776–778 (2011). 21764755 10.1126/science.1207745

[6] Wegner, D. M. Transactive memory: A contemporary analysis of the group mind. In *Theories of Group Behavior* 185–208 (Springer, 1987).

[7] Liang, D. W., Moreland, R. & Argote, L. Group versus individual training and group performance: The mediating role of transactive memory. *Personal. Soc. Psychol. Bull.* **21**(4), 384–393 (1995).

[8] Lewis, K. & Herndon, B. Transactive memory systems: Current issues and future research directions. *Organ. Sci.* **22**(5), 1254–1265 (2011).

[9] Henkel, L. A. Point-and-shoot memories: The influence of taking photos on memory for a museum tour. *Psychol. Sci.* **25**(2), 396–402 (2014). 24311477 10.1177/0956797613504438

[10] Ward, A. F. Supernormal: How the internet is changing our memories and our minds. *Psychol. Inq.Bold">24*(4), 341–348 (2013).

[11] Wegner, D. M. & Ward, A. F. How Google is changing your brain. *Sci. Am.* **309**(6), 58–61 (2013). 24383365 10.1038/scientificamerican1213-58

[12] Klein, S. B. Making the case that episodic recollection is attributable to operations occurring at retrieval rather than to content stored in a dedicated subsystem of long-term memory. *Front. Behav. Neurosci.* **7**, 3 (2013). 23378832 10.3389/fnbeh.2013.00003 PMC3561741

[13] Schacter, D. L., Addis, D. R. & Buckner, R. L. The prospective brain: Remembering the past to imagine the future. *Nat. Rev. Neurosci.* **8**, 657–661 (2007). 17700624 10.1038/nrn2213

[14] Suddendorf, T. & Corballis, M. C. The evolution of foresight: What is mental time travel and is it unique to humans? *Behav. Brain Sci.* **30**, 299–313 (2007). 17963565 10.1017/S0140525X07001975

[15] Tulving, E. Episodic memory and autonoesis: Uniquely human? In *The Missing Link in Cognition* (eds Terrace, H. S. & Metcalfe, J.) 3–56 (Oxford University Press, 2005).

[16] Schooler, C. Use it—And keep it, longer, probably: A reply to Salthouse (2006). *Perspect. Psychol. Sci.* **2**(1), 24–29 (2007). 26151916 10.1111/j.1745-6916.2007.00026.x

[17] Tucker-Drob, E. M. & Salthouse, T. A. Cognitive aging. In *The Wiley-Blackwell Handbook of Individual Differences* 242 (2011).

[18] University of Essex, Institute for Social and Economic Research. Understanding Society: waves 1–11, 2009–2020 and Harmonised BHPS: waves 1–18, 1991–2009. [data collection]. 15th Edition. UK Data Service. SN: 6614, (2022). 10.5255/UKDA-SN-6614-16

[19] McFall, S. Understanding society: UK household longitudinal study: Cognitive ability measures (2013). https://www.understandingsociety.ac.uk/wp-content/uploads/documentation/user-guides/6614-user-guide-cognitive-ability-measures.pdf.

[20] Dunnett, C. W. A multiple comparison procedure for comparing several treatments with a control. *J. Am. Stat. Assoc.* **50**(272), 1096–1121 (1955).

[21] Firth, J. et al. A meta-review of lifestyle psychiatry: the role of exercise, smoking, diet and sleep in the prevention and treatment of mental disorders. *World Psychiatry* **19**(3), 360–380 (2020). 10.1002/wps.20773 PMC7491615 32931092

[22] Dong, G. & Potenza, M. N. Behavioural and brain responses related to internet search and memory. *Eur. J. Neurosci.* **42**(8), 2546–2554 (2015). 26262779 10.1111/ejn.13039

[23] Dong, G. & Potenza, M. N. Short-term internet-search practicing modulates brain activity during recollection. *NeuroscienceBold">335*, 82–90 (2016). 27555549 10.1016/j.neuroscience.2016.08.028

[24] Liu, X. et al. Internet search alters intra-and inter-regional synchronization in the temporal gyrus. *Front. Psychol.* **9**, 260 (2018). 29559939 10.3389/fpsyg.2018.00260 PMC5845706

[25] Kanai, R., Bahrami, B., Roylance, R. & Rees, G. Online social network size is reflected in human brain structure. *Proc. R. Soc. B Biol. Sci.* **279**(1732), 1327–1334 (2012). 10.1098/rspb.2011.1959 PMC3282379 22012980

[26] He, Q., Turel, O., Brevers, D. & Bechara, A. Excess social media use in normal populations is associated with amygdala-striatal but not with prefrontal morphology. *Psychiatry Res. Neuroimaging*. **269**, 31–35 (2017). 28918269 10.1016/j.pscychresns.2017.09.003

[27] Montag, C. et al. Facebook usage on smartphones and gray matter volume of the nucleus accumbens. *Behavi. Brain Res.* **329**, 221–228 (2017). 10.1016/j.bbr.2017.04.035 28442353

[28] Inostroza, M. & Born, J. Sleep for preserving and transforming episodic memory. *Annu. Rev. Neurosci.* **36**, 79–102 (2013). 23642099 10.1146/annurev-neuro-062012-170429

[29] Erickson, K. I. et al. Physical activity, cognition, and brain outcomes: A review of the 2018 physical activity guidelines. *Med. Sci. Sports Exerc.* **51**(6), 1242 (2019). 10.1249/MSS.0000000000001936 PMC6527141 31095081

[30] Tremblay, M. S. et al. Canadian 24-hour movement guidelines for the early years (0–4 years): An integration of physical activity, sedentary behaviour, and sleep.*BMC Public Health* **17**(5), 1–32 (2017). 10.1186/s12889-017-4859-6 PMC5773896 29219102

[31] Andersson, G., Carlbring, P. & Hadjistavropoulos, H. D. Chapter 21—Internet-based cognitive behavior therapy. In *The Science of Cognitive Behavioral Therapy* (eds Hofmann, S. G. & Asmundson, G. J. G.) 531–549 (Academic, 2017). 10.1016/B978-0-12-803457-6.00021-0.

[32] Aboujaoude, E., Salame, W. & Naim, L. Telemental health: A status update. *World Psychiatry*. **14**(2), 223–230 (2015). 26043340 10.1002/wps.20218 PMC4471979

[33] Linardon, J., Cuijpers, P., Carlbring, P., Messer, M. & Fuller-Tyszkiewicz, M. The efficacy of app‐supported smartphone interventions for mental health problems: A meta‐analysis of randomized controlled trials. *World Psychiatry*. **18**(3), 325–336 (2019). 31496095 10.1002/wps.20673 PMC6732686

[34] Torous, J. & Firth, J. The digital placebo effect: Mobile mental health meets clinical psychiatry. *Lancet Psychiatry*. **3**(2), 100–102 (2016). 26851322 10.1016/S2215-0366(15)00565-9

